# Expanding the toolbox for *Trypanosoma cruzi*: A parasite line incorporating a bioluminescence-fluorescence dual reporter and streamlined CRISPR/Cas9 functionality for rapid *in vivo* localisation and phenotyping

**DOI:** 10.1371/journal.pntd.0006388

**Published:** 2018-04-02

**Authors:** Fernanda Cristina Costa, Amanda Fortes Francisco, Shiromani Jayawardhana, Simone Guedes Calderano, Michael D. Lewis, Francisco Olmo, Tom Beneke, Eva Gluenz, Jack Sunter, Samuel Dean, John Morrison Kelly, Martin Craig Taylor

**Affiliations:** 1 Faculty of Infectious and Tropical diseases, London School of Hygiene and Tropical Medicine, London, United Kingdom; 2 Institute of Physics of São Carlos, University of São Paulo, São Carlos, Brazil; 3 Laboratory of Parasitology, Instituto Butantan, São Paulo, Brazil; 4 Sir William Dunn School of Pathology, University of Oxford, Oxford, United Kingdom; 5 Department of Biological and Medical Sciences, Oxford Brookes University, Oxford, United Kingdom; Instituto de Investigaciones Biotecnológicas, ARGENTINA

## Abstract

**Background:**

Infection with *Trypanosoma cruzi* causes Chagas disease, a major public health problem throughout Latin America. There is no vaccine and the only drugs have severe side effects. Efforts to generate new therapies are hampered by limitations in our understanding of parasite biology and disease pathogenesis. Studies are compromised by the complexity of the disease, the long-term nature of the infection, and the fact that parasites are barely detectable during the chronic stage. In addition, functional dissection of *T*. *cruzi* biology has been restricted by the limited flexibility of the genetic manipulation technology applicable to this parasite.

**Methodology/Principal findings:**

Here, we describe two technical innovations, which will allow the role of the parasite in disease progression to be better assessed. First, we generated a *T*. *cruzi* reporter strain that expresses a fusion protein comprising red-shifted luciferase and green fluorescent protein domains. Bioluminescence allows the kinetics of infection to be followed within a single animal, and specific foci of infection to be pinpointed in excised tissues. Fluorescence can then be used to visualise individual parasites in tissue sections to study host-parasite interactions at a cellular level. Using this strategy, we have been routinely able to find individual parasites within chronically infected murine tissues for the first time. The second advance is the incorporation of a streamlined CRISPR/Cas9 functionality into this reporter strain that can facilitate genome editing using a PCR-based approach that does not require DNA cloning. This system allows the rapid generation of null mutants and fluorescently tagged parasites in a background where the *in vivo* phenotype can be rapidly assessed.

**Conclusions/Significance:**

The techniques described here will have multiple applications for studying aspects of *T*. *cruzi* biology and Chagas disease pathogenesis previously inaccessible to conventional approaches. The reagents and cell lines have been generated as a community resource and are freely available on request.

## Author summary

5–8 million people in Latin America are infected with the single-cell parasite *Trypanosoma cruzi*, the causative agent of Chagas disease. Of these, approximately one-third will develop chronic disease pathology, leading to disability and premature death. Only two drugs are available, both of which can have severe side effects. We know relatively little about the chronic phase of this infection because the parasite burden is extremely low, and animal models have limitations. Here, we describe a *T*. *cruzi* reporter strain that has been genetically modified to express a fusion protein which is both bioluminescent and fluorescent. These parasites can be monitored throughout the infection, and individual parasites identified in tissue sections from infected mice. This allows us for the first time to analyse host-parasite interactions at a cellular level in the chronic phase of infection. We have also incorporated a streamlined version of the CRISPR/Cas9 genome editing system into the reporter strain. We demonstrated the utility of this system by generating null mutants in the flagellar attachment protein GP72, by replacing the green fluorescent protein with a red fluorescent protein, and by tagging the endogenous protein DNA topoisomerase 1A. This *T*. *cruzi* reporter line will significantly enhance our ability to unravel the pathogenesis and immunology of chronic Chagas disease.

## Introduction

*Trypanosoma cruzi* is a protist parasite that causes Chagas disease in humans. An estimated 5–8 million people are infected, of whom approximately 30–40% will go on to develop severe, disabling and life-threatening pathologies of the heart and/or gastrointestinal system. Approximately 99% of those infected are thought to be unaware of their situation and are therefore currently untreated [1]. Whilst previously restricted to Central and South America, Chagas disease is emerging across the world due to migration. In addition, it is now recognised as being endemic in the southern United States from California to Georgia [2]. To date there are only two drugs available for treatment of Chagas disease, benznidazole and nifurtimox. The same parasite enzyme (the type I nitroreductase, TcNTR-1) activates both drugs and cross-resistance may arise [3]. Both drugs also exhibit severe side effects in a significant number of patients, leading to non-compliance, particularly since the dosing regimen averages 60–90 days [4, 5]. Consequently, there is a need for new drugs and for more research into vaccine development. However, a lack of understanding of parasite biology, interaction with the host, immune evasion strategies and the pathogenesis of the disease, severely impairs both drug and vaccine development programmes. *T*. *cruzi* infections are life-long despite robust CD8^+^ T-cell mediated responses [6, 7]. Hence, to survive the parasite must establish a niche where it is tolerated, ignored or undetected by the host [8]. Understanding this parasite persistence is key to both drug and vaccine development, and is likely to be a crucial event in pathogenesis [9].

The complex interactions of *T*. *cruzi* with the mammalian host, particularly in the chronic phase of infection, suggest that analysing parasite phenotypes in tissue culture systems will be of limited predictive value. Thus, there is a need to enhance small animal models so that virulence, pathogenesis and drug susceptibilities of the parasite can be manipulated and studied *in vivo*. The major issue with animal models of Chagas disease is that parasite numbers are both very low, and focally distributed during the chronic phase of infection, and they are rarely detectable in the blood [8, 10]. Therefore, it is difficult to establish correlates of cure, protection or disease progression. PCR-based detection using blood samples can be negative in infected individuals due to the focal and dynamic nature of the infection, and the technique is unable to distinguish living and dead organisms. Recently, we developed highly sensitive *in vivo* imaging models for both *T*. *cruzi* and the related parasite *T*. *brucei* (the agent of human African trypanosomiasis, or sleeping sickness) using a red-shifted variant (*PpyRE9h*) of the firefly luciferase gene to confer bioluminescence on the parasites [8, 11]. These models allow for the rapid and sensitive *in vivo* testing of new compounds or vaccines, such that the parasite burden can be followed in real time by monitoring the bioluminescence signal within an infected mouse [12–14]. These models can also be used to examine infection kinetics/dynamics of mutant parasites *in vivo* [15]. However, a major limitation of bioluminescence is that it is not easily adaptable for examining individual parasites at a cellular level.

Here, we describe methodology that has allowed us to generate *T*. *cruzi* that express bioluminescent-fluorescent fusion proteins. These dual-expresser parasites can be readily detected within chronically infected mouse tissue, so that as few as one amastigote can be identified in a tissue section. We also report the incorporation into these parasites of a T7 RNA polymerase/CRISPR/Cas9 system [16] in which genome editing can be achieved without the necessity of cloning the targeting donor DNA, or the single guide RNA (sgRNA) template DNA fragments. This model has the potential to accelerate research into the biology and pathogenesis of Chagas disease.

## Methods

### Parasite culture

*T*. *cruzi* CL-Brener epimastigotes were cultured in supplemented RPMI-1640 as described previously [17]. Genetically manipulated lines were routinely maintained on their selective agent (hygromycin, 150 μg ml^-1^; puromycin, 5 μg ml^-1^; blasticidin, 10 μg ml^-1^; G418, 100 μg ml^-1^). Tissue culture trypomastigotes (TCTs) were derived by infecting L6 rat myoblast cells with stationary phase metacyclic trypomastigotes. L6 cells were maintained in RPMI-1640/10% FBS without hemin/trypticase supplementation. Cell cultures were infected for 18 hours, external parasites were then removed by washing in medium, and the flasks incubated with fresh medium for a further 5–7 days. Extracellular TCTs were isolated by removal of the medium and centrifugation at 1600 *g*. Pellets were re-suspended in Dulbecco’s PBS and motile trypomastigotes counted using a haemocytometer.

### Construct preparation

**Fig 1:**
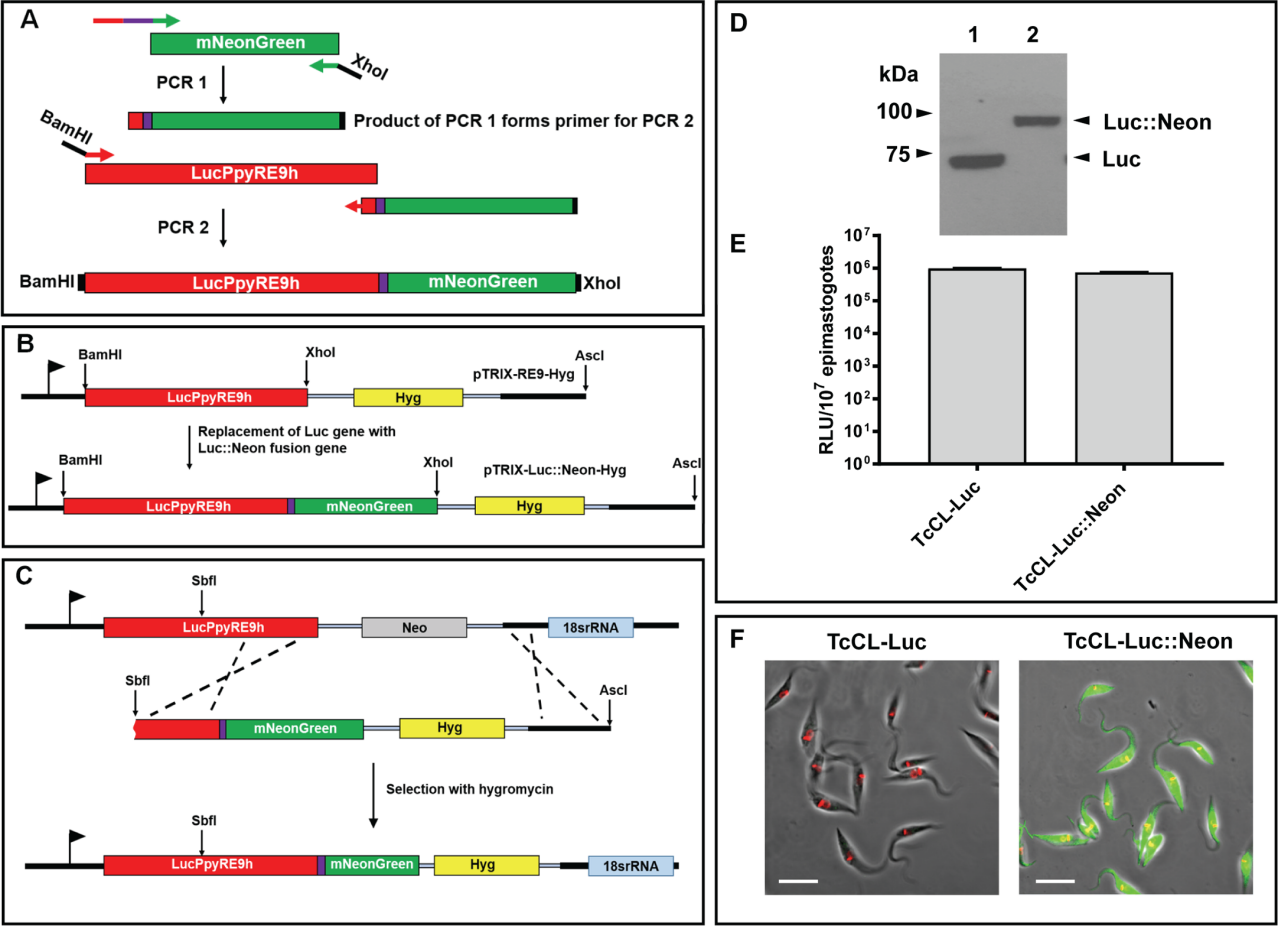
Generation and expression in *T*. *cruzi* of a chimeric bioluminescent–fluorescent fusion protein. **A.** Amplification of the mNeonGreen ORF and flanking targeting sequences, together with the coding sequence for an 8 amino-acid spacer peptide (purple line) (Methods) (PCR1). The product was used as the downstream primer to amplify the Luc::Neon fusion fragment (PCR2). The purple box indicates the 8 amino acid spacer peptide. **B.** The PCR2 product was inserted into plasmid pTRIX2-RE9-Hyg [15] to create pTRIX2-Luc::Neon-Hyg. **C.** SbfI/AscI linearised pTRIX2-Luc::Neon-Hyg was transfected into *T*. *cruzi* CL-Luc such that integration replaces the original luciferase sequence with the fusion gene, with the G418 resistance gene (Neo) exchanged for the gene encoding hygromycin resistance (Hyg). **D.** Western blot probed with a polyclonal anti-luciferase antibody (Promega). Lane 1, *T*. *cruzi* CL-Luc, the original bioluminescent strain; Lane 2, *T*. *cruzi* CL-Luc::Neon parasites expressing the 88 kDa fusion protein. **E.** Epimastigote luciferase activity in parasite lysates (Methods). Extracts were assayed in quadruplicate, data show mean activity, and error bars indicate standard deviation. **F.** Epimastigotes fixed in 2% paraformaldehyde and imaged on a Zeiss LSM 510 confocal microscope. The bar indicates 10μm. Only cells carrying the fusion protein exhibit green fluorescence. DNA is stained with DAPI (coloured red on images).

The *PpyRe9h-mNeonGreen* fusion gene was generated using a PCR-based method (Fig 1A). Firstly, the mNeonGreen gene was amplified from the plasmid pPOTv4 blast-blast mNeonGreen using the primers Luc::Neon Fus and mNeon Rev (Supplementary S1 Table; the sequence of pPOTv4 blast-blast mNeonGreen is available from http://www.sdeanresearch.com/resources/vector-sequences/archived-vector-sequences). The product of this reaction was then used as the reverse primer in conjunction with the forward primer Luc FWD. This reaction generated a 2.3 kb band comprising the fused genes, which was digested with BamHI and XhoI and ligated into similarly digested pTRIX2-RE9h-HYG to replace the 1.6 kb *PpyRE9h* gene with the *PpyRe9h-mNeonGreen* fused gene (Fig 1B). This resulted in the vector pTRIX2-Luc::Neon-HYG. For transfection of the bioluminescent CL-Brener strain (*T*. *cruzi* CL-Luc) [8], the plasmid was cut at an SbfI site within the luciferase ORF and at an AscI site downstream of the 3’ rRNA array targeting sequence. When transfected, the fragment integrated into the previously inserted luciferase coding sequence, also replacing the existing G418 resistance gene with a hygromycin resistance gene (Fig 1C).

To isolate the hSpCas9 nuclease gene [18], the pTrex-b-NLS-hSpCas9 plasmid (a gift from Rick Tarleton, University of Georgia) was first digested with XhoI, and then blunt-ended with Klenow DNA polymerase. The linearised plasmid was cut with XbaI to liberate the coding sequence [19], which was ligated into XbaI/AleI digested pLEW13 [20], to replace the *tet* repressor gene. The final construct (pLEW-Cas9) contained the T7 RNA polymerase, neomycin phosphotransferase and *cas9* genes, flanked by a β-tubulin targeting sequence from *T*. *brucei*. This sequence shares 89% nucleotide identity with the corresponding *T*. *cruzi* region and has previously been demonstrated to facilitate targeted integration in *T*. *cruzi* [21]. For transfection, the plasmid was linearised with NotI, which cleaves within the β-tubulin targeting sequence.

pPOTcruziv1 blast-blast mNeonGreen was based on the previously developed *T*. *brucei* tagging vectors with the *T*. *brucei* intergenic sequences replaced with *T*. *cruzi* equivalents [22]. Plasmid sequence data are available in the supplementary information.

### Parasite transfection

a) mNeonGreen tagging of PpyRe9h, and T7 RNA polymerase/Cas9 integration

Epimastigotes were transfected using a Nucleofector, with human T-cell buffer (Lonza) and program X-014. After nucleofection, parasites were transferred to 50 ml medium and incubated overnight at 27°C. The selective agent was then added (hygromycin, 150 μg ml^-1^ for pTRIX2-Luc::Neon-Hyg; G418, 100 μg ml^-1^ for pLew13-Cas9) and the parasites plated into 48 well plates. Plates were incubated at 27°C under 5% CO_2_. Positive wells were picked into culture flasks for further characterisation.

b) single guide RNA transcription templates and homology donors

sgRNA targeting sequences were designed using the “Eukaryotic Pathogen CRISPR guide RNA/DNA Design Tool” (EuPaGDT) software at http://grna.ctegd.uga.edu [23]. For amplification of sgRNA templates, 2 μM each of primer: CRISPR reverse (sgRNA scaffold) and a gene-specific forward primer, were mixed in 1×Phusion Master Mix (ThermoScientific), in 20 μl total volume. PCR steps were 30 s at 98°C, followed by 35 cycles of 10 s at 98°C, 30 s at 60°C, 15 s at 72°C, as described previously [16]. To amplify protein tagging or gene replacement cassettes, 30 ng circular pPOT, pPOTcruzi, pTbBLAST or pT plasmid DNA as appropriate, 2 μM each of gene-specific forward and reverse primer, and 3% (v/v) DMSO were mixed in 1×Phusion Master Mix, in 40 μl total volume. PCR steps were 5 min at 98°C, followed by 40 cycles of 30 s at 98°C, 30 s at 65°C, 2 min 15 s at 72°C, followed by a final elongation step for 7 min at 72°C. Homology donor targeting sequences of 30 bp per flank were designed by manual inspection of the appropriate sequence in TriTrypDB (http://tritrypdb.org/tritrypdb/) [24]. Generation of homology donors containing the blasticidin resistance gene required the cassette to be amplified from pNATx^TAG^-BSD [25] using primers pBlast_Tub_Swa_Fw and pBlast_Act_Swa_Rev. It was cloned into pJET1.2 (ThermoFisher Scientific) to create pTbBLAST, since donors derived from the pT-Blast vector [16] did not produce transformants. The pTbBLAST template carried RNA processing signals derived from the *T*. *brucei* tubulin and actin genes, which we had previously used in *T*. *cruzi* [15].

For transfection, the guide RNA transcription templates and the homology donor were mixed (20 μl per guide RNA template and 40 μl homology donor, using standard reaction volumes above). The DNA was purified using the PCR Clean-up kit (Qiagen) and eluted in 50 μl elution buffer. This purification step is necessary when using Phusion Master Mix, as there is a detergent present in the buffer which can lyse trypanosomes. In a total volume of 250 μl (50 μl DNA mix and 200 μl Tb-BSF buffer [26]), 10^7^ cells were transfected with the combined DNA mix in 0.2 mm gap cuvettes, using the Nucleofector program X-014. After nucleofection, parasites were transferred to 10 ml medium and incubated overnight at 27°C. The selective agent was then added (puromycin, 5 μg ml^-1^_;_ blasticidin, 10 μg ml^-1^) and the parasites plated out in 24 well plates in serial dilution.

### Quantification of *in vitro* luciferase activity

*In vitro* luciferase activity was measured using the luciferase assay system (Promega), following the manufacturer’s instructions. The cell culture lysis reagent (CCLR) was supplemented with 10 mg ml^-1^ bovine serum albumin. Parasites in exponential growth phase were pelleted and washed once with PBS, then lysed in 1xCCLR/BSA. Luciferase activity was measured in a Gemini plate reader at 610 nm (Molecular Devices).

### Mouse infection and necropsy

Mice were maintained under specific pathogen-free conditions in individually ventilated cages. They experienced a 12 hour light/dark cycle and had access to food and water *ad libitum*. Female mice aged 8–12 weeks were used. CB17 SCID mice were infected with 1x10^4^ tissue culture trypomastigotes, and monitored by bioluminescence imaging (BLI), as previously reported [8]. At the peak of the bioluminescence signal, when trypomastigotes were visible in the bloodstream, the mouse was sacrificed and the infected blood removed. The trypomastigotes were washed in Dulbecco’s PBS and diluted to 5x10^3^ ml^-1^. 1x10^3^ trypomastigotes were injected i.p into each BALB/c mouse and the course of infection followed by BLI. At specific time-points, the mice were euthanised and necropsied (for detailed description of the necropsy method, see Supplementary Information S1 Text.). Their organs were subject to BLI. We excised those segments that were bioluminescence-positive and placed them into histology cassettes. In each case, the heart tissue was fixed regardless of the BLI signal. When required, immunosuppression was carried out by the administration of three doses of cyclophosphamide (200 mg kg^-1^ i.p., Sigma-Aldrich) at 3–4 day intervals [13]. Mice were closely monitored for side effects from cyclophosphamide treatment or for secondary infections due to immunosuppression. BLI images from living animals and *post-mortem* tissues were analysed using Living Image 4.5.4 (PerkinElmer Inc.)

### Ethics statement

All animal work was performed under UK Home Office licence 70/8207 and approved by the London School of Hygiene and Tropical Medicine Animal Welfare and Ethical Review Board. All protocols and procedures were conducted in accordance with the UK Animals (Scientific Procedures) Act 1986 (ASPA). Mice were anaesthetised using 3% isofluorane by inhalation. Euthanasia was carried out by intraperitoneal injection of Euthatal (Pentobarbital Sodium).

### Tissue embedding and sectioning

To preserve the fluorescence of the Luc-mNeonGreen fusion protein, we adapted a method previously published for eGFP [27]. Excised tissue was fixed in pre-chilled 95% ethanol for 20–24 hours in histology cassettes. The tissues were then dehydrated using four changes of 100% ethanol over a total of 1 hour. They were cleared in xylene; two changes of 12 min each, and then embedded in paraffin at 56°C, two changes of 12 min each. Sections were cut with a microtome and mounted on glass slides, then dried overnight. Slides were stored in the dark at room temperature until the day before microscopic examination. For imaging, slides were deparaffinised in two changes (30 s each) of xylene, three changes (1 min each) of pre-chilled 95% ethanol, and three changes (1 min each) of pre-chilled Tris-buffered saline (TBS). Slides were mounted with vectashield/DAPI (VectorLabs) and stored at 4°C in dark until required.

### TUNEL assay for kDNA replication

Epimastigotes in logarithmic growth phase were fixed in suspension with 2% paraformaldehyde in PBS and air-dried onto glass 8-well slides. Cells were washed once in PBS and permeabilized in 0.1% TritonX-100/PBS for 5 mins and washed 3 times with PBS. 20 μL TUNEL reaction mixture (In situ Cell Death Detection Kit, TMR-red, Roche) was added to each well and the reaction was incubated for 1 hour at 37°C. The slides were washed three times in PBS and mounted in Vectashield with DAPI. Slides were examined by confocal microscopy.

### Confocal microscopy

Sections were examined using a Zeiss LSM510 Axioplan confocal laser scanning microscope. Cells containing multiple parasites were imaged in three dimensions (z-stacking) to allow precise counting of amastigotes (using the 63x or 100x objectives with appropriate scan zoom for the particular cell/number of parasites). Phase images were obtained at lower magnification (40x) to allow orientation of the tissue section and identification of specific layers/structures. All images were acquired using Zeiss LSM510 software. Scale bars were added using the Zeiss LSM Image Browser overlay function and the images were then exported as .TIF files to generate the figures. Movies were created either from the z-stack itself or using the projection utility of the Zeiss LSM Image Browser with z-stack source files.

## Results

### Generation of parasites expressing a bioluminescent-fluorescent fusion protein

Bioluminescence is useful for monitoring *T*. *cruzi* infection kinetics at a whole animal level, and for characterising tissue tropism *post mortem*, but it is less suitable for microscopic studies due to the requirement for D-luciferin and ATP and the transient nature of the light emission. To circumvent this, we fused a green fluorescent protein gene to a copy of a red-shifted luciferase already integrated in the *T*. *cruzi* genome, to allow expression of a polypeptide containing both activities. This approach would ensure that every bioluminescent parasite was also fluorescent. mNeonGreen was selected for use on the basis of its high fluorescence and photostability [28].

A fusion cassette was generated using an overlapping PCR strategy and incorporated into the pTRIX2-RE9h-Hyg plasmid (Methods, Fig 1A and 1B), replacing the *PpyRE9h* luciferase gene [15] such that the encoded N-terminal domain comprised the red-shifted luciferase and the C-terminal domain the mNeonGreen fluorescent protein. An eight amino-acid spacer peptide (TAGPGSAT) was incorporated between the luciferase and mNeonGreen sequences to minimise steric events that might disrupt correct folding of the domains. The plasmid was used to transfect the bioluminescent CL-Brener clone *T*. *cruzi* CL-Luc, such that it would integrate into the luciferase gene and confer hygromycin resistance in place of G418 resistance (Fig 1C, [8]). This approach was based on exploiting the high-level expression properties of the pre-tagged rRNA locus. Previous work in both *T*. *cruzi* and *T*. *brucei* had demonstrated that although expression can vary significantly between different rRNA coding arrays, the expression levels of individual loci are remarkably stable [8, 25, 29]. This suggested that this specific locus should ensure high-level expression of the fusion protein. We also engineered the construct to integrate into the rRNA loci of wild type strains.

Expression of the Luc::Neon fusion protein was analysed by western blotting using an anti-luciferase antibody, by assaying luciferase activity, and by examining cells for green fluorescence. The blots showed a single band, which migrated at the expected ~88 kDa molecular mass, compared to the original luciferase protein ~60 kDa (Fig 1D). Parasites expressing the fusion protein had similar levels of luciferase activity to the *T*. *cruzi* CL-Luc clone (Fig 1E), indicating that incorporation of the fluorescent protein sequence did not inhibit luciferase activity. Fluorescence microscopy demonstrated uniform green fluorescence throughout the parasite population (Fig 1F).

### Parasites expressing the fusion protein display a similar infection profile and tropism to the bioluminescent CL-Brener strain

**Fig 2:**
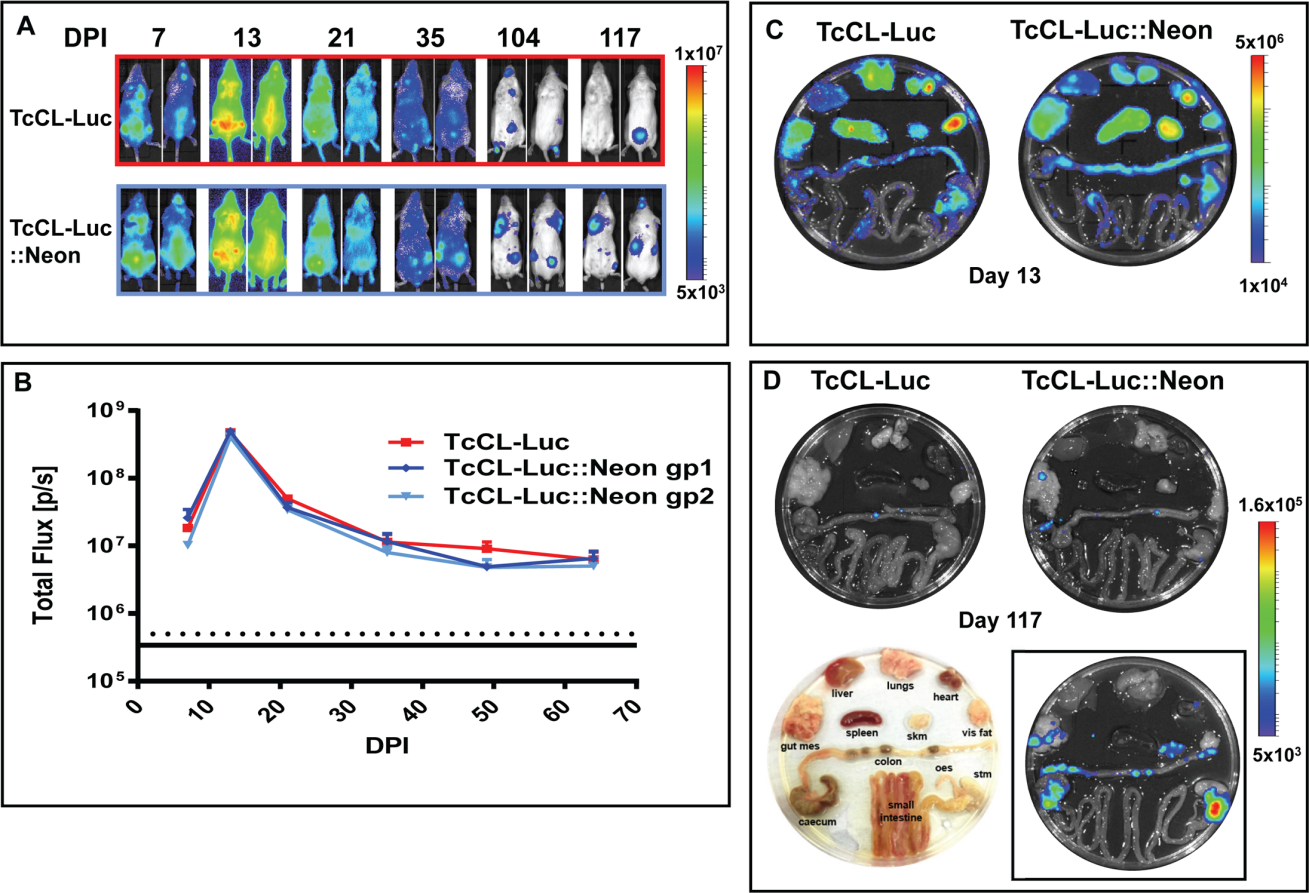
Expression of the dual reporter does not impinge on the infection profile. **A.** Bioluminescence imaging of representative infected mice (ventral and dorsal images shown) following i.p. infection with 1x10^3^ bloodstream trypomastigotes; upper panel, infection with the original *T*. *cruzi* CL-Luc clone; lower panel, infection with the *T*. *cruzi* CL-Luc::Neon clone. DPI; days post-infection. **B.** The total body flux for each group in photons/second (p/s). Data points represent the sum of the total flux from both dorsal and ventral views. Red line, group infected with *T*. *cruzi* CL-Luc; Blue lines, two individual groups infected with *T*. *cruzi* CL-Luc::Neon (n = 3 per group). Error bars represent SEM. **C.** *Post mortem* imaging of organs from mice in the acute phase of infection (day 13). **D.** Organs imaged in the chronic phase of infection (day 117). The image in the lower panel (left) indicates the arrangement of tissues. The image inset in the lower panel (right) shows organs from the chronic phase of infection (day 117) from a mouse immunosuppressed with cyclophosphamide (Methods), that was infected with *T*. *cruzi* CL-Luc::Neon. All images in D are calibrated to the same scale as indicated by the side bar.

To examine the effect of expressing the fusion protein on parasites *in vivo*, we infected female BALB/c mice with 10^3^ bloodstream trypomastigotes using our usual infection procedure [8, 10, 12, 13, 15, 16]. Simultaneously, mice were infected with the original bioluminescent strain, *T*. *cruzi* CL-Luc. We imaged the animals at several time-points to check the progress of the infection (Fig 2A). On the basis of bioluminescence, mice infected with the dual reporter strain were indistinguishable from those infected with the original *T*. *cruzi* CL-Luc strain, suggesting that expression of the fusion protein did not have a significant effect on either parasite growth or dissemination (Fig 2B).

Mice infected with the dual-expresser parasites were necropsied at 2 time-points; day 13, representing the peak of the acute phase, and day 117 representing an established chronic infection. During each necropsy, organs were imaged and those areas displaying a bioluminescent signal were excised, and fixed for microscopic analysis. Tissue dissemination by parasites expressing the fusion protein was similar to that in the original *T*. *cruzi* CL-Luc strain. At day 13, most organs examined showed a significant signal, whereas by day 117, bioluminescence was largely restricted to the gastro-intestinal tract (GIT) (Fig 2C and 2D). Immunosuppression with cyclophosphamide resulted in an increased signal in the GIT and the appearance of bioluminescent foci in other tissues (Fig 2D). Thus, expression of the fusion protein did not significantly affect the capacity of the parasites to establish a chronic phase GIT niche, as demonstrated previously in this model, and did not restrict the ability of the parasites to proliferate and disseminate following immunosuppression with cyclophosphamide [8].

### Fluorescent parasites can be readily identified in ethanol-fixed, paraffin-embedded tissue sections

**Fig 3:**
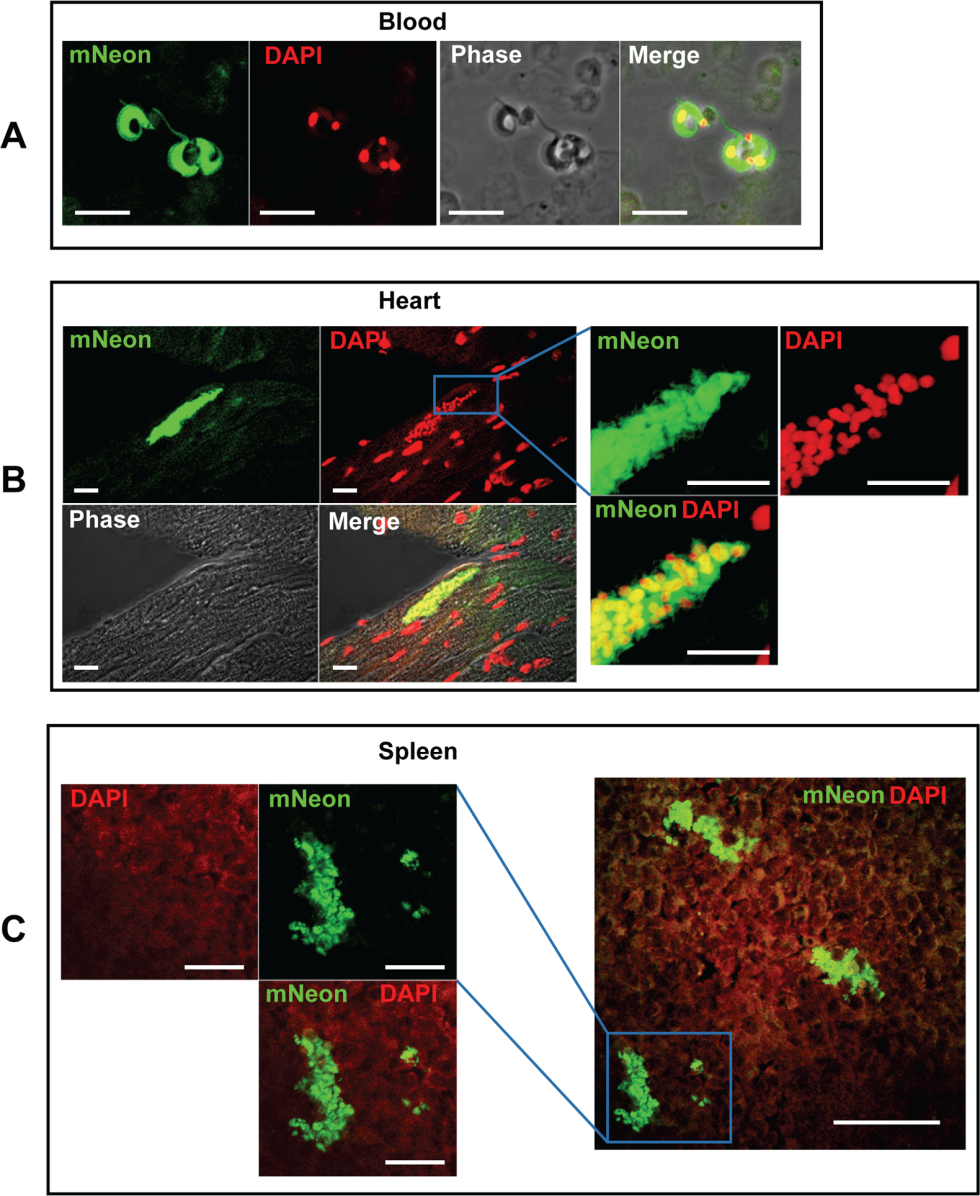
Fluorescent detection of parasites in the bloodstream, heart and spleen of mice during the acute stage of infection. **A.** Fluorescent trypomastigotes observed in the bloodstream on day 13 post-infection. Blood was fixed with 2% paraformaldehyde in PBS and examined by fluorescence microscopy. Bar indicates 10 μm. **B.** A parasite nest in cardiac muscle (day 13). The right-hand panel shows the boxed area at higher magnification. Bar indicates 10 μm in both sets of images. **C.** Parasite clusters in the spleen from the same mouse as in B. Some of these parasites appear to be damaged, suggesting that they may be under immunological attack. The left-hand panel shows the boxed area at higher magnification; the bar indicates 20 μm. In the right-hand panel, the bar represents 50 μm.

We fixed bioluminescent segments of tissue from necropsied animals in 95% ethanol for 24 hours and then embedded them in paraffin wax. This fixation protocol has been demonstrated previously to preserve the fluorescence of eGFP and mTomato [27]. Sections were cut, placed on slides, rapidly deparaffinised, rehydrated and then examined by confocal microscopy (Methods). Tissues examined included heart, adipose tissue, colon, cecum, stomach, skeletal muscle and spleen in the acute phase and heart, colon, cecum, and stomach in the chronic phase. If another organ displayed bioluminescence in a particular mouse, then that was also taken for analysis. In addition, blood samples were taken. In the acute stage (day 13), mNeonGreen fluorescence was visible in all tissue types examined and parasite location readily identifiable at a cellular level. Fluorescent extracellular trypomastigotes were also detectable in the blood (Fig 3A), indicating that the reporter fusion protein persisted in these non-dividing parasites, which have decreased ribosomal RNA transcription [30]. In addition, large nests of parasites were visible in sections of heart tissue (see for example, Fig 3B and supplementary S1 Movie). In the spleen of the same animal, we observed clusters of parasites, in which some displayed aberrant morphology consistent with exposure to immune attack (Fig 3C).

**Fig 4:**
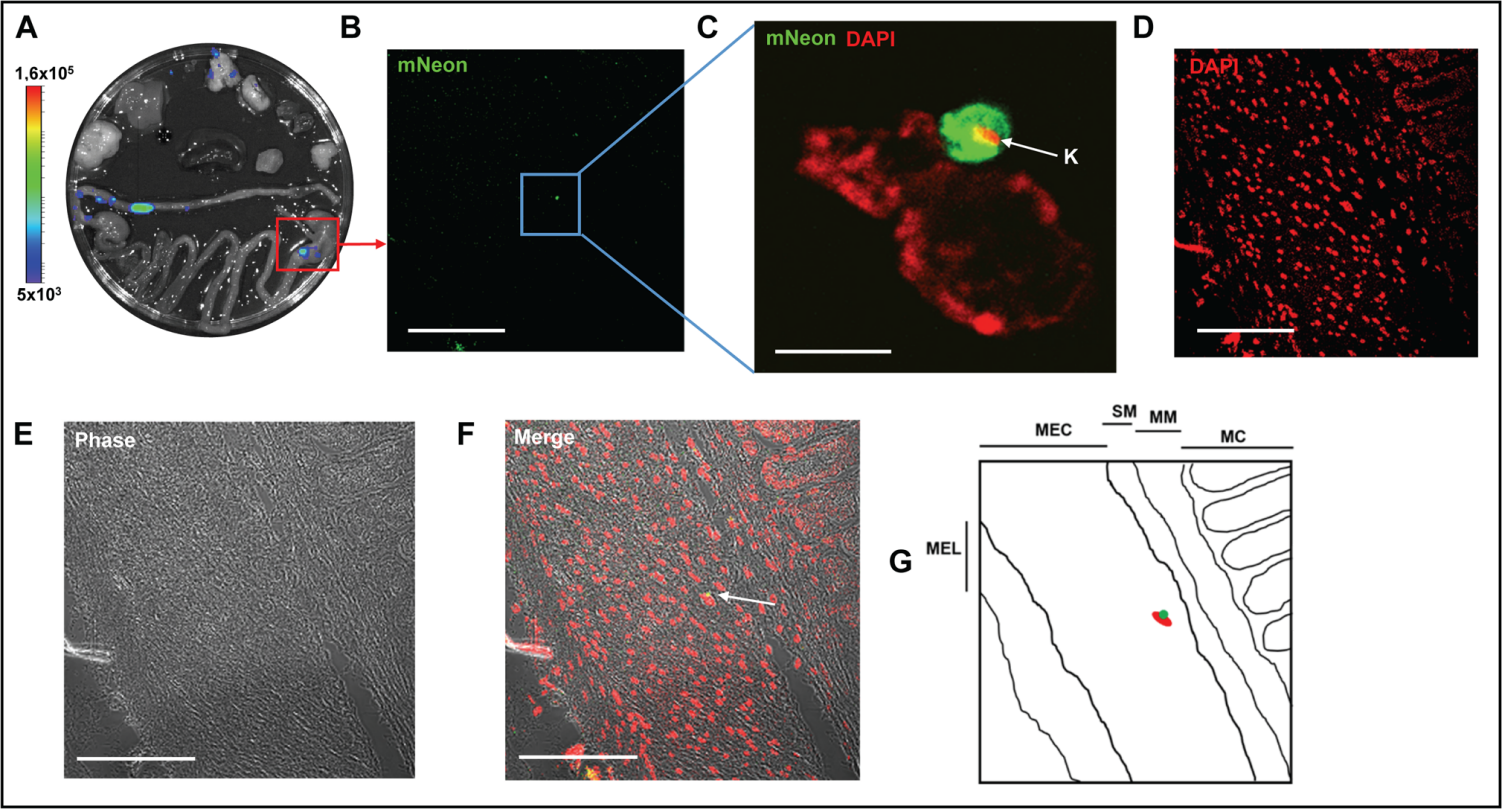
A single amastigotes can be identified in a whole tissue section during chronic stage infections. **A.** *Ex vivo* bioluminescence image of organs from a chronically infected mouse (day 117), showing parasite presence in stomach, colon and lungs (organs placed as in Fig 2D). The red box identifies the tissue sample from which the section shown in B-F was taken. **B.** mNeonGreen fluorescence. **C.** Higher magnification of boxed area in B, showing a single amastigote in close conjunction with the nucleus of the host cell. The kinetoplast DNA (K) is indicated by an arrow. **D.** DNA staining of section shown in B. **E.** Phase image illustrating architecture of tissue section. **F.** Merged image of B, D and E, showing phase, DNA and mNeonGreen. The single amastigote is indicated by an arrow and appears yellow. For panel C, bar represents 5 μm; for all other panels, bar indicates 100 μm. Images B, D, E and F are taken with a 40x objective at 0.7 scan zoom, image C is taken with 100x objective, scan zoom 2.7. **G.** Schematic indicating individual layers of the section. MC, mucosa; MM, muscularis mucosae; SM, submucosa; MEC, muscularis externae (circular); MEL, muscularis externae (longitudinal). The approximate location of the infected cell is indicated within this schematic (red oval and green circle).

**Fig 5:**
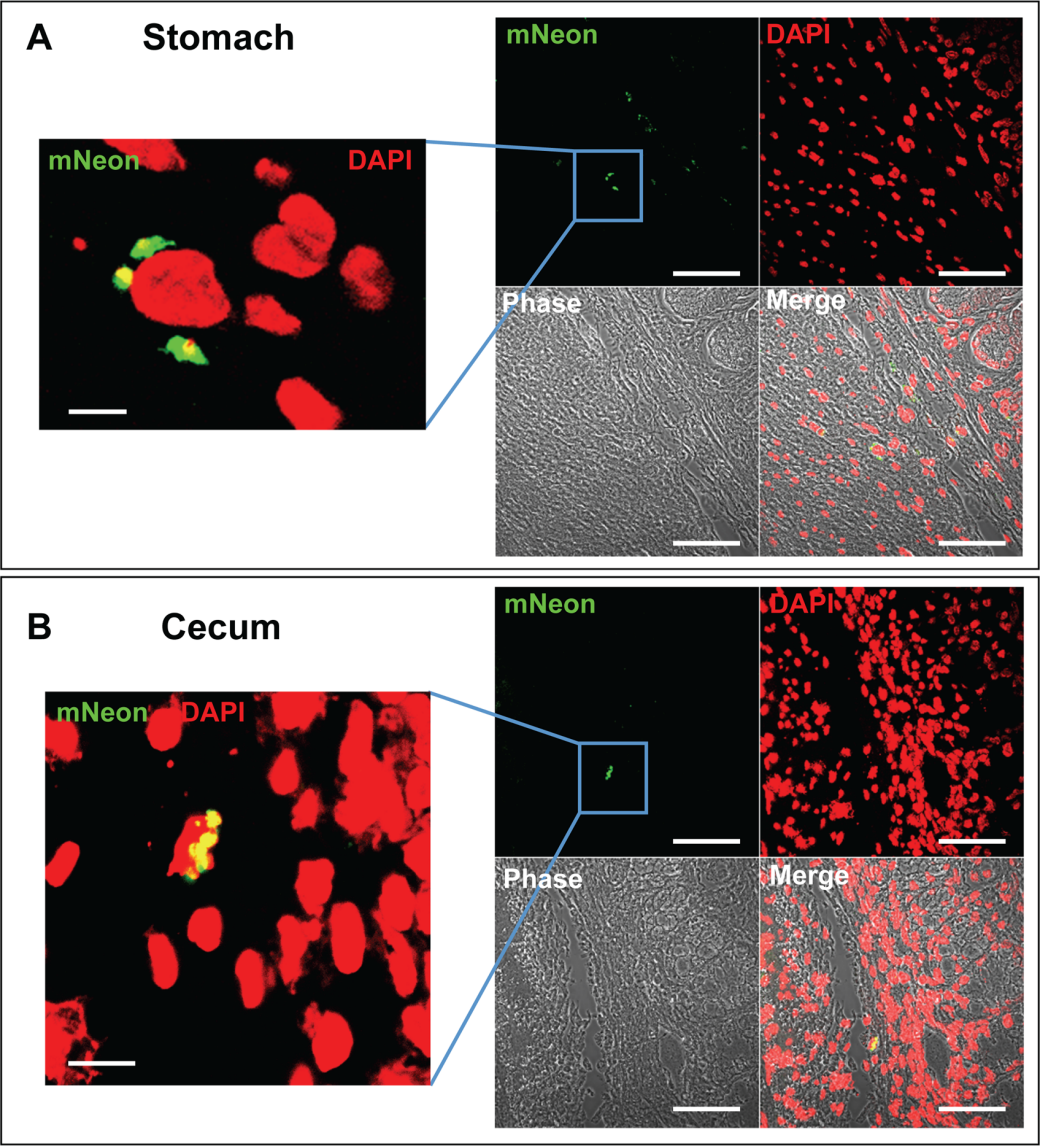
Location of parasites in the stomach and cecum during chronic stage infections. **A.** Section of stomach tissue from a chronically infected mouse (day 117)—a single slice from a Z-stack through the infected cell (63x scan zoom 0.7). The left-hand panel is a magnified view of the boxed section (63x scan zoom 2.8). The bars represent 5 μm (left hand panel) and 50 μm (right hand panel). **B.** Section of cecum tissue from a chronically infected mouse (day 117), with a cluster of amastigotes surrounding the nucleus of an infected cell (left hand image, 63x scan zoom 2.5; right hand image, 63x scan zoom 0.7).

When we examined tissues from the chronic phase (day 117 post-infection), significantly fewer parasites were detectable, as expected from the bioluminescent signal (compare Fig 2C and 2D, note the different logarithmic scale bars used). The process used to scan images is illustrated in Fig 4. Tissue is taken specifically from areas that are bioluminescence positive (Fig 4A). Firstly, we view each section under green fluorescence (Fig 4B, 40x objective, scan zoom 0.7). On locating a putative fluorescent parasite, we check the image for DAPI staining of kinetoplast and nuclear DNA–this often requires zooming-in, (for example in Fig 4C, 100x objective with 2.7 scan zoom) as DAPI staining of parasite DNA under low magnification can be eclipsed by the intense signal from the host cell nucleus (compare Fig 4C and 4D). False positive signals due to auto-fluorescent objects can be resolved at this stage, particularly in gut sections, where food-derived particles can often be fluorescent. We then scan the image at low magnification (40x objective, 0.7 scan zoom), so that both fluorescent and phase images can be captured (Fig 4D and 4E). The phase image allows the identification of different layers or structures within the sections. The parasite itself and surrounding tissue is then imaged at a higher magnification (Fig 4C, for example). The fluorescence of the mNeonGreen is bright enough to allow single amastigotes to be identified when scanning a tissue section of ~10 mm^2^ at 40x (Fig 4B, 4C and 4F). Where multiple co-infecting amastigotes are present, we take a z-stack of the infected cell/s to ensure that all parasites can be discriminated and counted (for example, supplementary S1 Fig and S2 Movie). Examples from other chronically infected gut sections are shown in Fig 5.

### Integration of a T7 RNA polymerase/Cas9 cassette into the dual reporter strain

**Fig 6:**
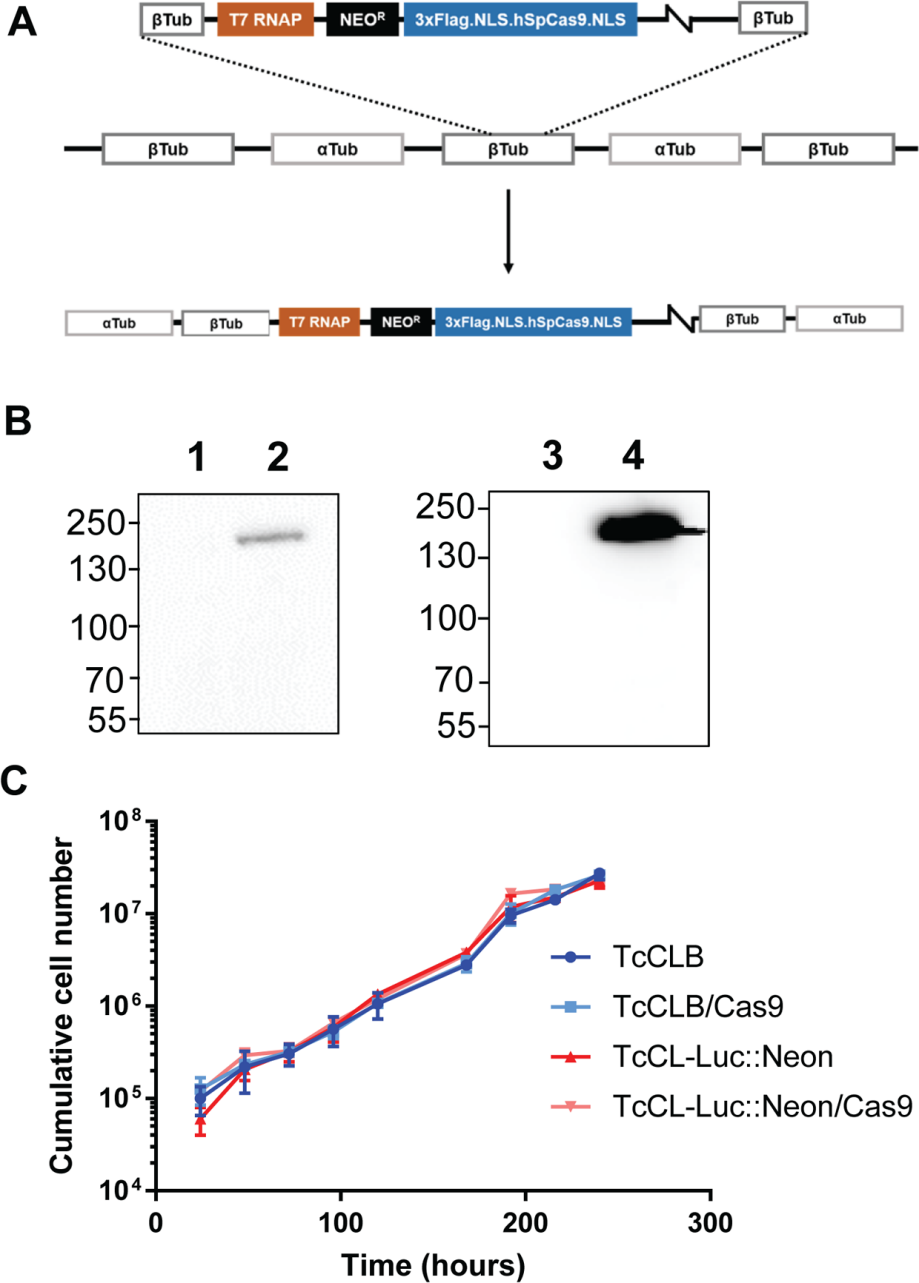
Incorporation of CRISPR/Cas9 functionality into the *T*. *cruzi* CL Luc::Neon reporter line. **A.** Map of pLEW13-Cas9 showing the construct and the integration of linearised DNA into the *T*. *cruzi* genome via the tubulin locus (Methods). **B.** Western blots showing expression of the *Cas9* gene. Wild type *T*. *cruzi* CL-Brener before (lane 1) and after (lane 2) transfection with pLEW13-Cas9. Right hand panel; *T*. *cruzi* CL-Luc::Neon reporter strain before (lane 3) and after (lane 4) transfection. The blot was probed with anti-Cas9 monoclonal 7A9 (Merck). **C.** Cas9 has no effect on growth when expressed from the tubulin array. Growth curves comparing both parental lines with their Cas9 expressing counterparts. Growth assays were performed in triplicate; the error bars represent standard deviation.

As illustrated above, the dual reporter strain can be exploited as a descriptive tool to explore *T*. *cruzi* infection, both *in vivo* and *ex vivo*. However, subsequent functional studies can be labour intensive because of the cumbersome nature of genetic manipulation procedures in *T*. *cruzi*. With this in mind, we incorporated the CRISPR/Cas9 system into the dual reporter strain. Several CRISPR/Cas9 configurations have been developed for kinetoplastids [16, 19, 31–35]. The T7 RNA polymerase/Cas9 system [16] seemed to offer the greatest ease and flexibility. Here, the guide RNA is transfected as a double-stranded DNA PCR product containing a T7 promoter at the 5’ end of the guide strand, and is transcribed *in vivo* in the target cell. The homology-directed repair templates, which are also generated by PCR and do not require any cloning step, are co-transfected with the guide template, with genomic modification events occurring within 48 hours of transfection, as the guide RNA template is subsequently lost [16]. To incorporate this system into the *T*. *cruzi* reporter strain, firstly the T7 RNA polymerase and Cas9 genes were integrated into the tubulin gene array (Fig 6A). In parallel, the two genes were also engineered into the wild type CL-Brener strain, to facilitate fluorescent protein tagging experiments, which would be confounded by expression of the mNeonGreen sequence. Expression of Cas9 was confirmed by western blotting (Fig 6B). Cas9 has previously been shown to have a detrimental effect on the growth of *T*. *cruzi* epimastigotes when expressed from a plasmid under the control of the strong rRNA promoter [19], although others have not observed this problem [31]. We therefore examined the growth rate of the Cas9 expressing lines. When expressed from the tubulin array, the Cas9 gene appears to have no significant effect on the growth rate of the parasites, in either wild type or *T*. *cruzi* CL-Luc::Neon backgrounds (Fig 6C).

### Demonstration of T7 polymerase/CRISPR/Cas9-mediated genetic manipulation functionality in *T. cruzi*

**Fig 7:**
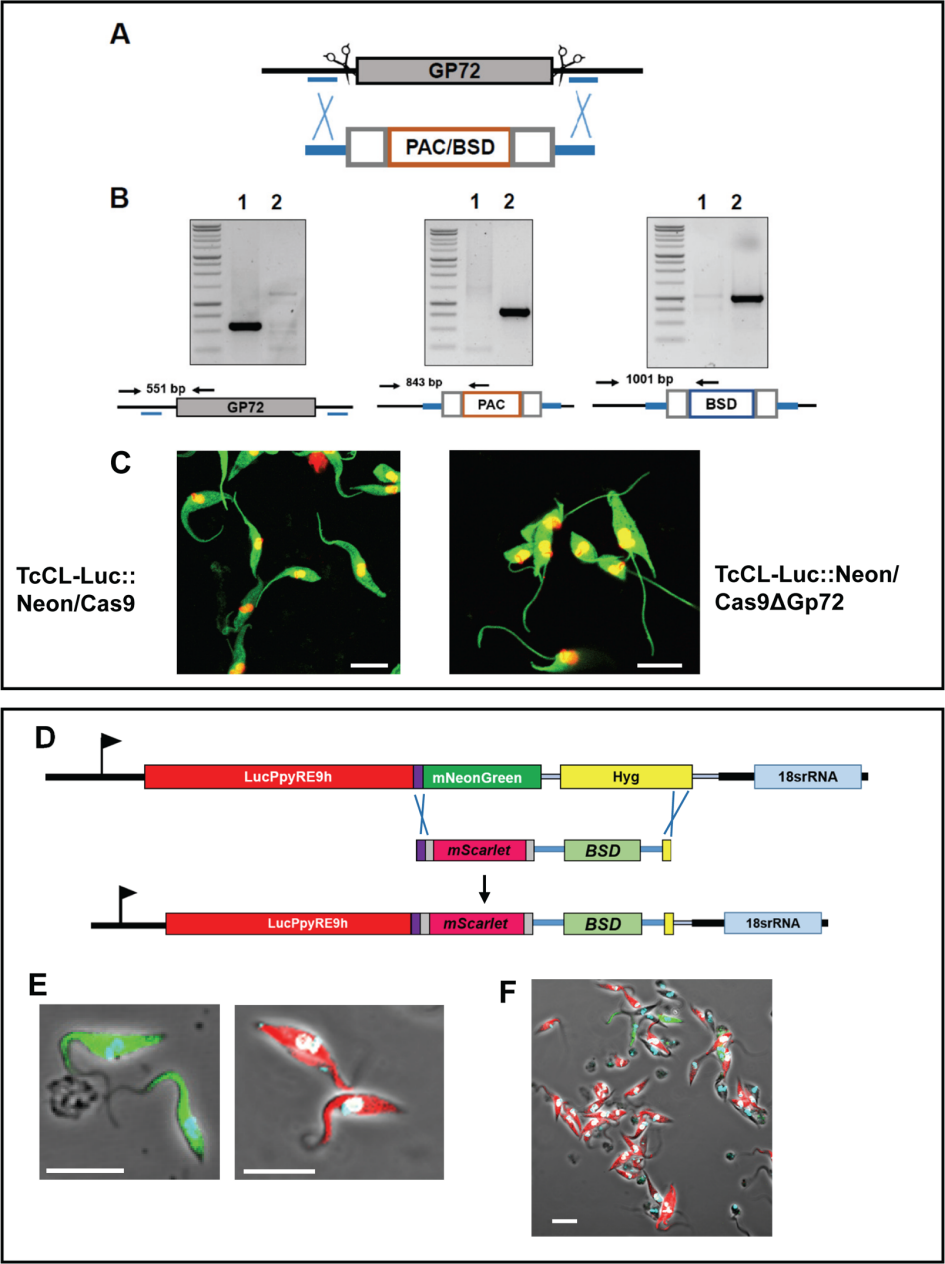
CRISPR/Cas9 mediated genome editing in the *T*. *cruzi* CL-Luc::Neon reporter strain. **A.** Diagram of the *GP72* gene indicating sgRNA binding sites (indicated by scissors) and homology arms for integration (blue bars). The lower map shows the configuration of the puromycin (PAC) and blasticidin (BSD) drug resistance cassettes. Grey boxes indicate plasmid derived RNA processing signals; orange box shows the coding sequence for the selectable marker. **B.** PCR-based confirmation of gene deletion (Methods). Lane 1, parental clone, Lane 2, a null mutant clone. The relative positions of the primers are illustrated on the maps below each panel. The *GP72* gene is only present in the parental clone and both selectable markers have integrated as expected. **C.** The *GP72* null mutant parasites are characterised by detachment of the flagellum from the cell body. Left hand panel, the parental strain (TcCL-LNCas9); right hand panel, the *GP72* null mutant. Note that the flagellum in the null mutants exits the cell immediately anterior to the kinetoplast, whereas in the parental strain it remains attached along the length of the cell body. **D.** Strategy for CRISPR-Cas9 mediated replacement of mNeonGreen by mScarlet. The map of the Luc::Neon locus indicates the Cas9/sgRNA mediated cleavage sites. The homologous repair donor is illustrated, with the 30 bp homology arms which correspond to the spacer peptide coding sequence and the 3’-end of the hygromycin resistance gene. The edited locus is shown in which the mScarlet and blasticidin resistance genes have replaced the mNeonGreen and hygromycin resistance genes. **E.** Images of parasites before (green) and after (red) replacement of the fluorescent reporters. Images taken with 100x objective at 2.3 scan zoom. **F.** Image of the parasite population under drug selection, 12 days after transfection, taken with 63x objective at 1.3 scan zoom. The majority of parasites now display red fluorescence. In all images, the white bars represent 10μm.

**Fig 8:**
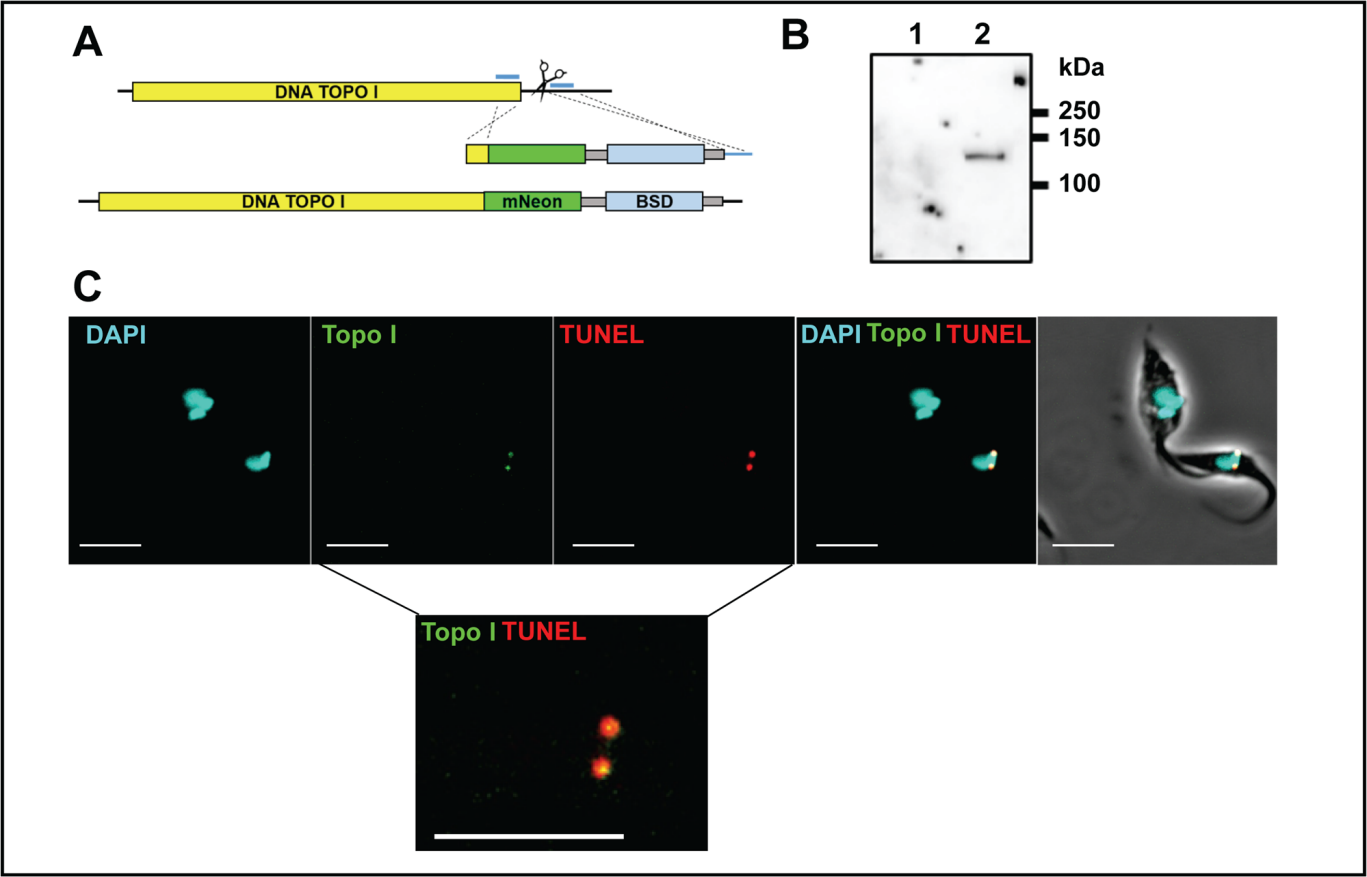
CRISPR/Cas9 mediated tagging and localisation of DNA topoisomerase 1A. **A**. Map of the DNA topoisomerase 1A gene. The single sgRNA/Cas9 cleavage site is indicated by scissors. Blue lines indicate the positions of sequences corresponding to the 30 bp homologous arms in the repair fragment. The lower image shows the edited gene containing the 3’ fusion with mNeonGreen and the position of the selectable blasticidin (BSD) resistance gene. Grey boxes indicate sequences required for mRNA processing. **B.** Western blot showing the expression of the tagged DNA topoisomerase 1A. Lane 1, the CL-cas9 parental strain with an untagged locus; Lane 2, mNeonGreen tagged cell line showing a single band of the expected size (120 kDa). The blot was probed with anti-mNeonGreen (ChromoTek GmbH) **C.** Images showing that fluorescence is restricted to antipodal sites on the kinetoplast in parasites expressing mNeonGreen tagged DNA topoisomerase 1A. These sites coincide with regions of TUNEL positivity (Methods). Note that the upper parasite in the image, where the kinetoplasts have replicated and segregated, is negative for both mNeonGreen and TUNEL staining. The lower inset shows a magnified image where the mNeonGreen/TUNEL staining patterns are merged. Co-localisation appears as yellow. The bar indicates 5μm.

To check that the T7 polymerase directed CRISPR/Cas9 system was functional in *T*. *cruzi*, we carried out several proof-of-concept manipulations, (i) deletion of both copies of *GP72* (TcCLB.509561.20), (ii) replacement of mNeonGreen with mScarlet in the cell line encoding the dual reporter fusion protein, and (iii) C-terminal tagging of DNA topoisomerase 1A with mNeonGreen (for sgRNA target sites, see supplementary information, S2 Table). Transfections (i) and (ii) were carried out on the *T*. *cruzi* CL-Luc::Neon/Cas9 strain, using two sgRNA templates cutting either side of the DNA to be deleted, while transfection (iii) was carried out on the *T*. *cruzi* CL-T7Pol/Cas9 strain using a single sgRNA (S2 Table, Figs 7 and 8, Methods). Drug resistant parasites cells were readily obtained in all cases.

(i) Deletion of *GP72*: The GP72 protein is the *T*. *cruzi* homologue of *T*. *brucei* Fla1, and is required for flagellar attachment [36, 37]. *GP72* null mutants have previously been generated using traditional technology, and have a characteristic morphology which is easily scored [38]. The homology-directed repair templates used targeting arms located upstream and downstream of the sgRNA recognition sites, such that these sites are deleted from the edited sequence and cannot be re-cleaved (Fig 7A). Both homology donors (PAC and BSD) were transfected at the same time. Double drug resistant cells were isolated within 3 weeks of transfection, compared to the usual 3–6 months required for these types of manipulations using traditional approaches. PCR analysis indicated that the *GP72* alleles had been deleted and replaced by the puromycin and blasticidin resistance markers (Fig 7B). The *GP72* null mutant cells displayed the expected flagellar detachment phenotype (Fig 7C [38]). In all cells, the flagellum exited the cell immediately adjacent to the kinetoplast, whereas in the parental clone, the flagellum remained attached to the anterior of the cell body.

(ii) *In vivo* replacement of mNeonGreen with mScarlet in the dual reporter fusion gene: Novel bioluminescent and fluorescent reporter proteins are constantly being developed. We therefore sought to demonstrate that the CRISPR/cas9 system could be used to swap reporter domains in the chimeric fusion protein gene directly within trypanosomes, bypassing the *E*. *coli* cloning step. The new red fluorescent protein mScarlet [39] was chosen to provide a straightforward test since the parasites would switch from green to red fluorescence. Guide RNA templates were designed corresponding to the spacer peptide sequence of the Luc::Neon fusion protein and to the 3’ end of the hygromycin resistance gene (Fig 7D). We designed the homology donor to integrate the mScarlet gene in-frame with the luciferase sequence, while preserving the spacer peptide. The homology donor (amplified from pPOTv7 blast-blast mScarlet) also included the blasticidin resistance gene, to replace the hygromycin resistance gene in the edited locus. Parasites were transformed with the sgRNA and mScarlet donor PCR products. Twelve days after transfection, the parasites were fixed and examined by fluorescence microscopy. Most parasites were now red rather than green indicating that the correct gene editing reaction had taken place (Fig 7E and 7F, S2 Fig).

(iii) Tagging DNA topoisomerase 1A with mNeonGreen: DNA topoisomerase 1A (TcCLB.506493.80) is a mitochondrial enzyme associated with kDNA replication in trypanosomes [40]. We transfected *T*. *cruzi* CL-T7Pol/Cas9 with an sgRNA template located between 92–112 base pairs downstream of the coding sequence and a homology donor generated using pPOTcruzi v1.Blast.mNeonGreen as a template. The homology flanks were at the 3’ end of the coding sequence, excluding the stop codon, and adjacent to the sgRNA guide (Fig 8A). This configuration incorporated the mNeonGreen gene into the 3’ end of the Top1A coding sequence resulting in a C-terminally tagged protein. The tagged protein was of the expected size, ~120 kDa (Fig 8B). Drug resistant cells only expressed mNeonGreen fluorescence in antipodal sites on the kinetoplast disk (Fig 8C). Moreover, expression of the tagged protein appeared to be limited to a subpopulation of cells. Replication of minicircles results in newly synthesised strands that maintain the breaks induced by RNA priming until all of the minicircles are replicated. This allows the replication status of a kDNA network to be assessed using TUNEL assays (Methods), in which terminal transferase labels nicks in the new circles with fluorescent dUTP analogues. The TcTop1AmNeonGreen co-localized with TUNEL labelled DNA, demonstrating that Top1A in *T*. *cruzi* localises to the antipodal sites, where the minicircles are re-attached to the growing network (Fig 8C).

## Discussion

The study of chronic Chagas disease has suffered from limitations in the available animal models. This has been primarily due to difficulties in locating and quantifying the burden of *T*. *cruzi* in a stage of the disease where very few parasites are present. A consequence of this is that immunological and other events in the vicinity of parasite-infected tissues and cells cannot easily be characterised. We have recently developed a highly sensitive bioluminescence model for *T*. *cruzi* infection, which circumvents some of these issues [8]. It allows the dynamics of infection, drug efficacy and the behaviour of null mutants to be examined [10, 12, 13, 15]. However, extension of these macro imaging studies to a cellular and microscopic level has been restricted by the ATP-dependent nature of the reaction that generates bioluminescence, since this requires live cells. To maximise the usefulness of the system for exploring parasite biology and disease pathogenesis, we have expanded the utility of the imaging model by generating parasites expressing a chimeric protein in which luciferase is fused to a bright, photostable monomeric fluorescent protein, mNeonGreen [28]. The fusion ensures co-expression of the two activities, so that every parasite that is bioluminescent will also be fluorescent (Fig 1F). Consequently, when a bioluminescent focus is excised from tissue and sectioned, it is now feasible to visualise the parasites *in situ*, even when the parasite burden is extremely low. For example, single amastigotes can be found relatively quickly within sections of infected tissue, so that it will be possible to compare events provoked by large nests of rapidly replicating *T*. *cruzi*, and those generated in response to cells containing single parasites. This will allow the precise nature of host cells during chronic infections to be defined, and their immediate immunological environment to be characterised. These central questions have been difficult to address with the previous technologies.

Genetic manipulation procedures for *T*. *cruzi* have lagged somewhat behind those of its close relative *T*. *brucei*. There are two major reasons for this: first, transfection of *T*. *brucei* bloodstream forms gives rise to clonal populations much more rapidly than is the case with *T*. *cruzi* epimastigotes (6 days versus 4–6 weeks); second, *T*. *brucei* possesses the molecular machinery for RNAi, rendering gene knockdown approaches very effective [41, 42]. *T*. *cruzi* lacks RNAi machinery [21] and, being diploid, therefore requires two rounds of transfection to create null mutants, and three rounds if a conditional rescue vector is required. We had previously shown that T7 RNA polymerase activity can be readily expressed in *T*. *cruzi* [43], while various CRISPR/cas9 systems have also been shown to be functional in this parasite [19, 31, 35].This suggested that incorporation of the T7 RNA polymerase/CRISPR/cas9 system should be a feasible and flexible approach to genome editing in this parasite. In line with this, we found that the time taken to create null mutants could be reduced to less than 1 month. Utilisation of independent drug resistance markers in the homology donors removes the need for parasite cloning as only null mutants will be selected. Since the procedure requires PCR products alone, does not necessitate cloning in *E*. *coli* or *in vitro* transcription, this makes the system much more amenable to, and scalable for, medium/high throughput screening. In addition, its incorporation into bioluminescent-fluorescent parasites allows for a seamless transfer of functional analysis from an *in vitro* to an *in vivo* context.

We demonstrated the functionality of the genome editing system by recapitulating the phenotype of a null mutant previously generated using conventional genetic modification methods. Deletion of both *GP72* alleles results in epimastigotes that exhibit a detached flagellum. Using the new system, we were able to isolate null mutants within 3 weeks of transfection, with all parasites exhibiting the expected flagellar detachment phenotype (Fig 7C). We also tested the system for protein tagging, as has been shown with other CRISPR/Cas systems used in *T*. *cruzi* [35, 44]. Firstly, we swapped the mNeonGreen portion of the reporter fusion for a red fluorescent protein (mScarlet), using a method that bypasses *E*. *coli* cloning. Secondly, in trypanosomes that did not express the fluorescent fusion protein, we tagged the mitochondrial DNA topoisomerase 1A protein with mNeonGreen. We were able to show localisation of mNeonGreen to the antipodal replicative sites. The tagged protein was only expressed in replicating kinetoplasts, as evidenced by TUNEL labelling.

The trypanosome lines reported here extend the utility of the murine model we have recently developed, by allowing the capture of microscopic, cellular and local host-parasite interaction data from each experimental animal, in addition to the infection kinetics and organ tropism. This will also result in a reduction of numbers of animals required, since more information can be gained from each mouse. The incorporation of a genome editing system further increases the utility of the parasites by rendering the reporter line amenable to rapid throughput genetic manipulation, opening the way to an *in vivo* interrogation of parasite virulence, drug resistance and immune evasion.

## Supporting information

S1 Text Detailed method for necropsy. (DOCX)

S1 Dataset Plasmid sequences. (TXT)

S1 Fig Assessment of amastigote numbers within an infected cell from a z-stack image. **A:** Left hand panel shows a phase image of an infected cell isolated from adipose tissue. The right hand panel shows the same cell with mNeonGreen and DAPI fluorescence overlaid indicating the relative position of parasites and the mammalian cell nucleus. **B:** 3 slices from a z-stack of the cell shown in A. The three panels show the fluorescent image of each slice. **C:** The same three panels as in B with each amastigote outlined in a different colour. **D:** The fluorescent staining has been darkened to visualise the individual outlines derived in C. From the image, it is clear that there are six amastigotes within this one cell. The bar indicates 5 μm. The z-stack is also presented in S2 Movie.
(TIF)

S2 Fig Parasites expressing mNeonGreen or mScarlet have mutually exclusive fluorescence. Mixed parasite population from the experiment shown in Fig 7 imaged in both red and green channels to show that fluorescence is only present in the appropriate channel for each protein. The bar indicates 10 μm.
(PPTX)

S1 Movie Z-stack projection in the x-axis of a parasite nest within the heart tissue of a BALB/c mouse at day 13 post infection. Red represents DAPI staining for host and parasite DNA, green is the mNeonGreen fluorescence of the parasites. Flagellated trypomastigotes are visible at the edge of the nest. The z-stack was acquired with 63X objective at a scan zoom of 2.0, with a Z-depth of 11.2 μm, on a Zeiss LSM510 confocal microscope.
(ZIP)

S2 Movie Z-stack of the infected cell depicted in S1 Fig. Red represents DAPI staining for host and parasite DNA, green is the mNeonGreen fluorescence of the parasites. The z-stack was acquired with 100X objective at a scan zoom of 2.8, with a Z-depth of 15 μm, on a Zeiss LSM510 confocal microscope.
(ZIP)

S1 Table Primer sequences used in this study. (XLSX)

S2 Table sgRNA target sites. (DOCX)
