# Supplementary material for: Expanding the toolbox for Trypanosoma cruzi: A parasite line incorporating a bioluminescence-fluorescence dual reporter and streamlined CRISPR/Cas9 functionality for rapid in vivo localisation and phenotyping
Source: PLoS Negl Trop Dis. 2018 Apr 2;12(4):e0006388. doi: 10.1371/journal.pntd.0006388 (PMC5897030; doi:10.1371/journal.pntd.0006388)
Supplement: S1 Text — (DOCX) [file pntd.0006388.s001.docx]

**Necropsy and *ex vivo* imaging protocol**

*Trypanosoma cruzi* is a highly infectious agent. All of the stages present in mouse blood and tissue should be considered infectious to human beings. There is no current limit for the infectious dose, but it is estimated that ONE parasite may be enough to initiate infection in an immunocompetent host. Therefore, it is vital that all procedures involving *ex-vivo* imaging, extraction of blood etc. are performed in a microbiological safety cabinet (Class II), and that all sharps precautions are meticulously observed. The biological hazard classification of *T. cruzi* varies between countries, but it is advisable to treat it as a BL3 agent when handling infected blood, tissues etc., until the parasites have been permanently inactivated.

**Materials:**

Set up the following materials inside the safety cabinet:

- - Doubled carcass bag, taped to the back of the safety cabinet wall
  - Rack for 50 ml tubes
  - Dulbecco’s PBS (D-PBS) in 50 ml tube in a rack
  - 50 ml dilute luciferin (0.3 mg ml^-1^) in D-PBS, in a 50 ml tube (50 ml D-PBS and 1 ml luciferin stock, 15 mg ml^-1^), protected from light. Tube is kept in rack.
  - 1 ml pastette kept in rack, along with tubes of D-PBS and dilute luciferin
  - 2 x 250 ml plastic beakers – one with virkon (or suitable equivalent) and another with 70% alcohol
  - Sharps bin
  - 1 large plastic container with a tablet of virkon, filled two thirds to the top with water (for disposal of contaminated plastics)
  - 2 ml syringes
  - P200 Pipette (or equivalent) and sterile tips
  - Wax dissection board with 4 green 21G needles stuck into it in the middle
  - Dissection instruments: scalpel attached to blade x2, gripper forceps for skin x1, small forceps x2, large forceps x2, small scissors x2, large scissors x1, pointed scraper to clear the inside of the colon/stomach.
  - 4 histology cassettes and 4 lids
  - 3MM Filter papers cut into quarters
  - Biopsy papers
  - Petri dishes
  - Optional: 24 well black culture dish (for extra *ex vivo* tissues)
  - paper towels, folded and torn into thirds to act as blotting paper
  - Spray bottle of 70% ethanol
  - Spray bottle of virkon (or equivalent disinfectant)
  - 10 ml syringe
  - Green 21G needles
  - 1 ml syringe
  - 1.5ml tube for blood (if required) labelled “BLD”

Set up the following outside the safety cabinet, on a trolley, to the left of the safety cabinet:

- - 95% ethanol in biological safety container
  - Histology cassettes (labelled in pencil along the front and side) and lids (4 per mouse)
  - Luciferin syringe (1 ml) with 26G needle (0.2 ml per mouse) Keep dark.
  - Euthatal syringe (1 ml) with 26G needle (0.2 ml per mouse)

Set up the following outside the safety cabinet,

Half a petri dish lined with circular filter paper, with small circular filter papers placed evenly across the surface.

- - Scales on top of which the lined petri dish is placed

**Method:**

Weigh the mouse to be analysed and record weight.

Perform standard *in vivo* imaging as required: ventral, dorsal

Allow the mouse to recover and return to cage for minimum 1 hour

Inject a second dose of luciferin (0.2 ml) i.p. route, place mouse in a spare cage

and leave for 5 minutes.

Inject 0.2 ml euthatal i.p.

Monitor animal and do not proceed until unresponsive to foot pinch

Pin out body on wax dissection board, with legs stretched out and pin with needles.

Cut away skin at the right arm pit and sever axial vessels, while holding the skin upwards and away from the axial vessels, to contain the pooled blood.

Collect the blood with a 2 ml syringe and transfer to the 1.5 ml ‘BLD’ tube. Note the syringe should NOT have a needle attached.

Pinch a bit of skin above the thorax using the gripper forceps and dissect skin down the midline, discarding the skin covering the abdomen/thorax region.

Cut upwards from the midline of the peritoneum and thorax, and across both sides from the diaphragm to expose the heart and inner thorax region.

Fill a 10 ml syringe with dilute luciferin in D-PBS and attach a green 21G needle.

Perfuse the heart via the left ventricle, using the 10 ml luciferin syringe, taking care not to pull the needle out until perfusion is complete.

Clean up excess fluid/blood with pieces of blue paper towel and discard into virkon.

Lay out two halves of a petri dish adjacent to one another and using a pastette, transfer the 2% luciferin into pre-assigned positions on the petri dish, corresponding to the layout of organs/tissues, as shown in Fig 1 below:


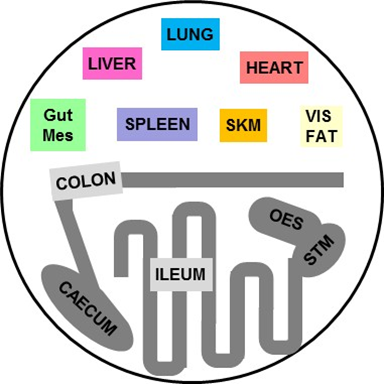


**Fig 1: Pre-assigned positioning of organs/tissues**

Dissect heart (blot any excess blood onto filter paper) and spleen from the carcass and place each of them on the small filter papers positioned in the petri dish on the scales.

Weigh each of them separately and record the weights.

Transfer the spleen to the petri dish into the allocated position shown in Fig 1, with the thicker end of the spleen to the left.

Place the heart on a filter paper, with the valves positioned upwards and slightly to the left.

Using the large forceps and scalpel, with one clean slice along the middle, cut the heart in half, and position the two halves adjacent to each other on the petri dish (at the position shown in Fig 1), exposing the internal structure, with the valves at the top.

Clean the scalpel blade with 70% ethanol and wipe clean (wipe on paper towel on floor of hood, DO NOT wipe with towel held in fingers).

Dissect the lungs, a sample of the liver (from the largest lobe), a small sample of visceral fat (from the gonadal deposit) and a sample of the skeletal muscle (from the left thigh) and position on the petri dish at the pre-assigned positions shown in Fig 1.

Carefully cut away the whole gut from the carcass, leaving as much of the oesophagus attached to the stomach as possible.

Carefully separate the mesenteries from the gut as it is removed, while simultaneously separating the stomach and caecum/colon.

Place the mesenteries at the position on the petri dish shown in Fig 1.

Position the colon along the petri dish as a straight horizontal line (as shown in Fig 1) and position the connected caecum in a ‘C’ or ‘S’ shape, whichever is best to avoid any kinks along the length.

Transfer the stomach/oesophagus/ileum to the petri dish (pre-assigned location shown in Fig 1) and position such that the free end of the ileum lies vertically on the petri dish and gently guide the length of the ileum into loops, positioned without gaps. Position the stomach so that it is placed slightly away from the ileum, with the oesophagus then placed in a hook position, away from the stomach, with all sections of the gut exposed as clearly as possible, as shown in Fig 2 below:


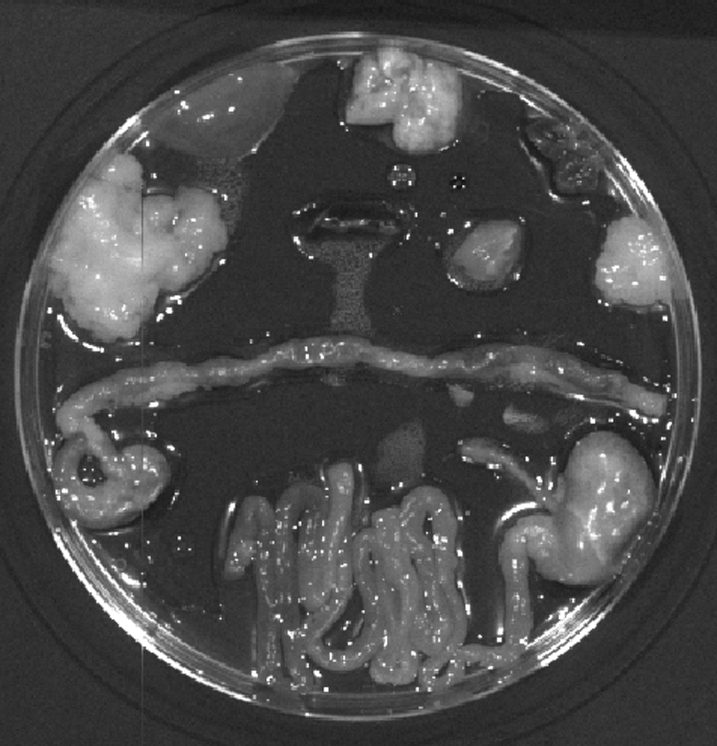


**Fig 2: Layout of organs/tissues on petri dish.**

Using the pastette, bathe the organs/tissues in dilute luciferin, taking care not to disrupt the layout of the organs/tissues.

Transfer the petri dish to the imaging chamber and position the petri dish as shown in Fig 2, and image for 5 minutes on large binning (IVIS Lumina II).

Transfer the remaining sections of the liver, visceral and subcutaneous fat, and ventral peritoneum and transfer into a petri dish alongside the carcass.

Position the carcass in the middle of the petri dish, with the skin cut away and peeled back, exposing as much of the internal cavity as possible.

Cut the skin along the thighs and arms and peel the skin back, exposing as much of the muscle as possible.

Cut the skin between the ears and the cheeks, sever the ear canal and peel the skin back to expose the internal regions of the neck/head. Spread the skin flat against the petri dish.

Flatten out the peritoneum and lay them to the right of the carcass, adjacent to each other.

Position the rest of the liver to the top left of the carcass, followed by the visceral fat to the middle left of the carcass, and the subcutaneous fat to the lower left of the carcass, as shown below in


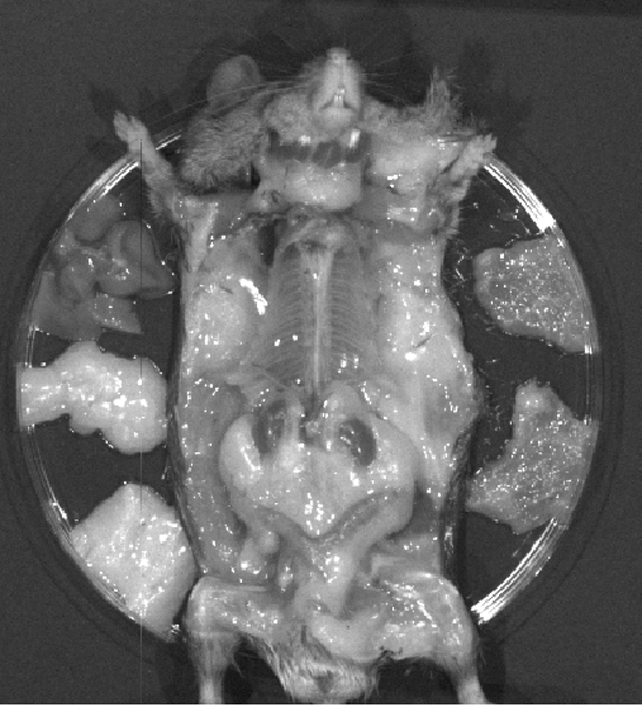


**Fig 3: Layout of carcass with remaining tissues on petri dish.**

Using the pastette, bathe the carcass/tissues in dilute luciferin, taking care not to disrupt the layout of the tissues.

Transfer the petri dish with the carcass to the imaging chamber and position the petri dish as shown in Fig 3, and image for 5 minutes on large binning.

Once the imaging of the organs/tissues is complete, transfer the petri dish back to the safety cabinet so that samples can be taken for histology and PCR.

Take a petri dish and pour in some D-PBS. All samples for histology and PCR must be washed in D-PBS prior to transferring to tubes/histology cassettes.

Samples for histology: Tissue pieces to be cut to appropriate size and placed into cassettes. Tissue sections are taken based on bioluminescent signal positivity except for the heart which is routinely taken regardless of signal.

**Cassette 1:** Heart and skeletal muscle

**Cassette 2:** Spleen and liver

**Cassette 3:** Gut: All pieces must be washed in D-PBS and cleared of the internal faeces onto a filter paper, using the small forceps and pointed scraper. Pieces of gut must then be laid out vertically along the length of the cassette and covered with a biopsy paper, before the lid is placed on top.

**Cassette 4:** Any other bioluminescent tissues

Ensure all histology cassettes are tightly closed, and immerse in the 95% ethanol for 24 hours at 4°C before being processed.

Transfer all unused body parts and contaminated paper to carcass bag, tie in a knot and dispose of according to local rules.

Transfer all contaminated liquids and plastic-ware to the large virkon plastic container, which is then emptied into the sink, with the plastic-ware inside discarded into solid-waste bins and the plastic box (and two small pots used for virkon and 70% ethanol) washed and left to dry.

Clean instruments with virkon and then soak in 70% ethanol, between working on different mice as well as at the end of the session, apart from scalpel blade, which is only cleaned with 70% ethanol between dissecting different mice.

Remove the blade from the scalpel holder WITH forceps and discard in the sharps bin.

Clean the safety cabinet with virkon and then 70% ethanol.
