# Supplementary figures and images for: Expanding the toolbox for Trypanosoma cruzi: A parasite line incorporating a bioluminescence-fluorescence dual reporter and streamlined CRISPR/Cas9 functionality for rapid in vivo localisation and phenotyping
Source: PLoS Negl Trop Dis. 2018 Apr 2;12(4):e0006388. doi: 10.1371/journal.pntd.0006388 (PMC5897030; doi:10.1371/journal.pntd.0006388)

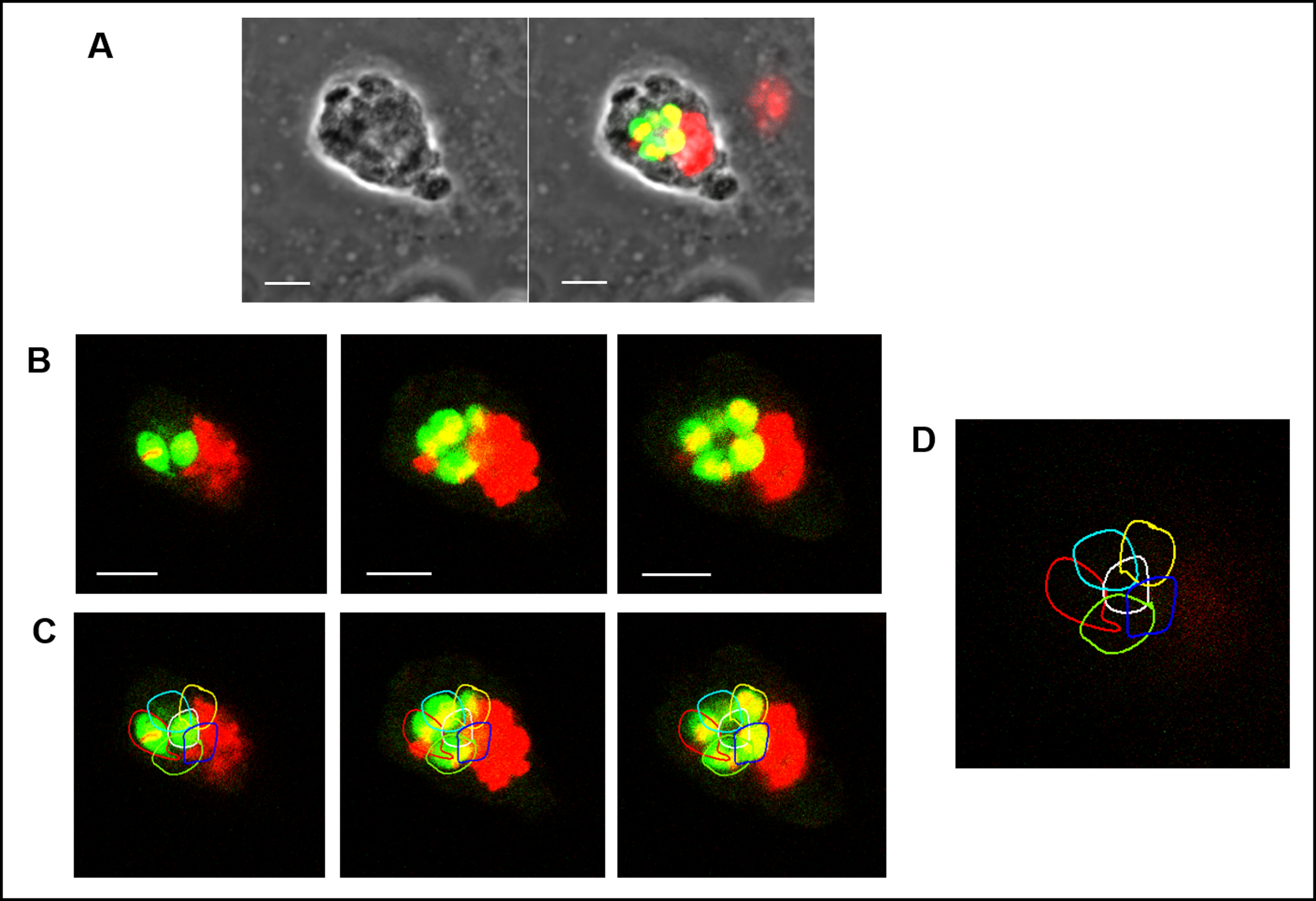

Supplement: S1 Fig — A: Left hand panel shows a phase image of an infected cell isolated from adipose tissue. The right hand panel shows the same cell with mNeonGreen and DAPI fluorescence overlaid indicating the relative position of parasites and the mammalian cell nucleus. B: 3 slices from a z-stack of the cell shown in A. The three panels show the fluorescent image of each slice. C: The same three panels as in B with each amastigote outlined in a different colour. D: The fluorescent staining has been darkened to visualise the individual outlines derived in C. From the image, it is clear that there are six amastigotes within this one cell. The bar indicates 5 μm. The z-stack is also presented in S2 Movie. (TIF) [file pntd.0006388.s003.tif]

## Slide 1
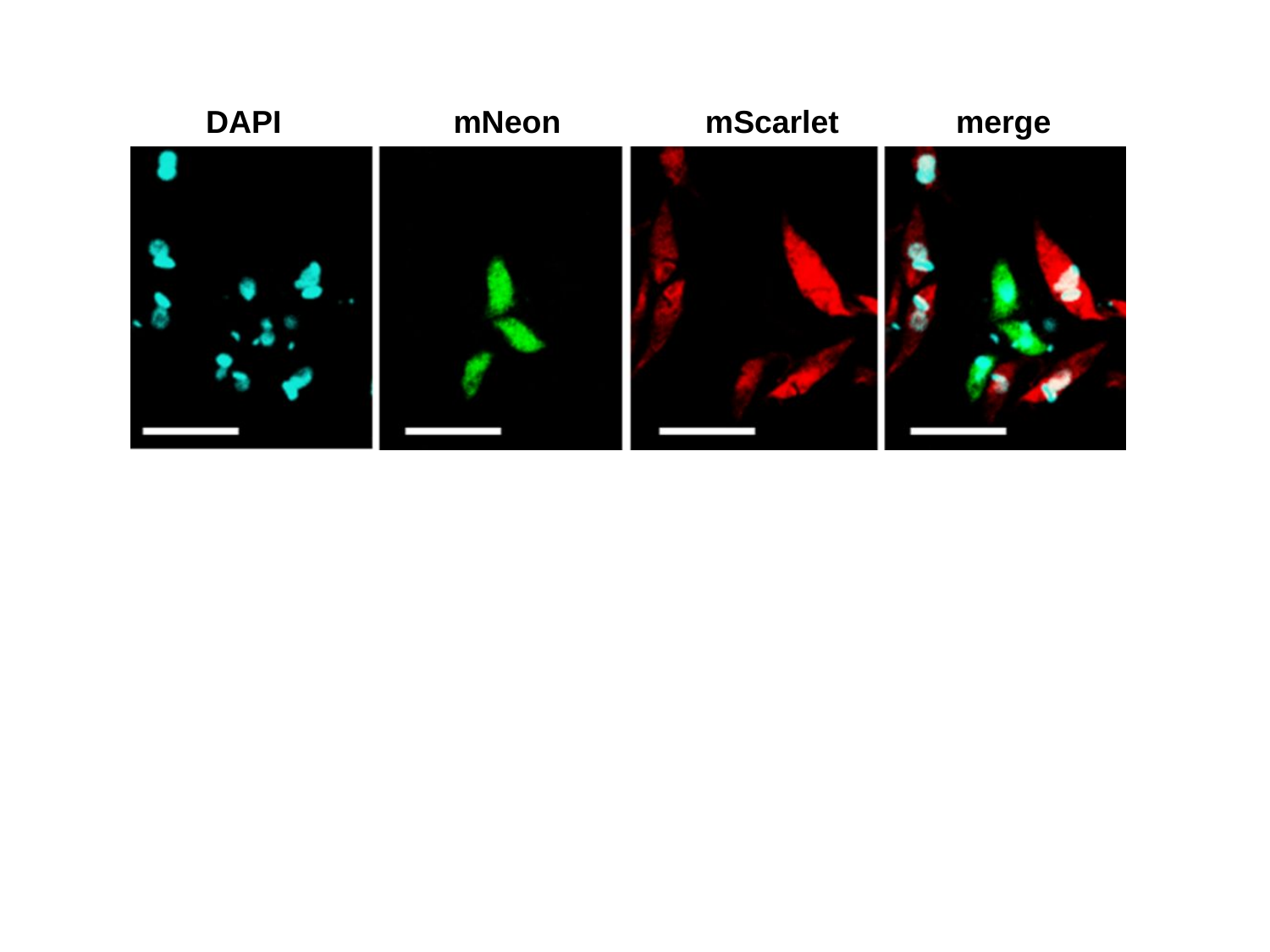

DAPI
mNeon
mScarlet
merge

Supplement: S2 Fig — Mixed parasite population from the experiment shown in Fig 7 imaged in both red and green channels to show that fluorescence is only present in the appropriate channel for each protein. The bar indicates 10 μm. (PPTX) [file pntd.0006388.s004.pptx]
