# Supplementary material for: Expanding the toolbox for Trypanosoma cruzi: A parasite line incorporating a bioluminescence-fluorescence dual reporter and streamlined CRISPR/Cas9 functionality for rapid in vivo localisation and phenotyping
Source: PLoS Negl Trop Dis. 2018 Apr 2;12(4):e0006388. doi: 10.1371/journal.pntd.0006388 (PMC5897030; doi:10.1371/journal.pntd.0006388)
Supplement: S2 Table — (DOCX) [file pntd.0006388.s008.docx]

| Target | Gene ID* | sgRNA 1  PAM  Location* | sgRNA 1 target sequence | sgRNA 2  PAM  Location* | sgRNA 2 target sequence |
| --- | --- | --- | --- | --- | --- |
| TcGP72 | TcCLB.509561.20 | -8 | GTAGACCGGACGTCCCGTCT | +1835 | GCAAGCGAAGGTGAAAACAA |
| Luc::Neon | N/A | +1748 | ATTTGGGTCGATCAACGGTG | +4072 | GAGTACTTCTACACAGCCAT |
| DNA topo1A | TcCLB.506493.80 | +2599 | TTATTATTATCCTTCGGGTG | N/A | N/A^#^ |

*PAM location mapped relative to A of the ATG of the CDS being +1

^#^ Only one guide RNA is used for endogenous tagging of proteins
